# An effective encoding of human medical conditions in disease space provides a versatile framework for deciphering disease associations

**DOI:** 10.1002/qub2.93

**Published:** 2025-03-11

**Authors:** Tianxin Xu, Yu Li, Xin Gao, Andrey Rzhetsky, Gengjie Jia

**Affiliations:** ^1^ Genome Analysis Laboratory of the Ministry of Agriculture and Rural Affairs Agricultural Genomics Institute at Shenzhen Chinese Academy of Agricultural Sciences Shenzhen China; ^2^ Department of Computer Science and Engineering (CSE) The Chinese University of Hong Kong (CUHK) Hong Kong China; ^3^ Computer Science Program Computer, Electrical and Mathematical Sciences and Engineering Division King Abdullah University of Science and Technology (KAUST) Thuwal Saudi Arabia; ^4^ Center of Excellence for Smart Health (KCSH) King Abdullah University of Science and Technology (KAUST) Thuwal Saudi Arabia; ^5^ Center of Excellence on Generative AI King Abdullah University of Science and Technology (KAUST) Thuwal Saudi Arabia; ^6^ Department of Medicine Institute of Genomics and Systems Biology Committee on Genomics, Genetics, and Systems Biology University of Chicago Chicago Illinois USA; ^7^ Department of Human Genetics University of Chicago Chicago Illinois USA

**Keywords:** biomedical data mining, disease embedding, machine learning

## Abstract

It is challenging to identify comorbidity patterns and mechanistically investigate disease associations based on health‐related data that are often sparse, large‐scale, and multimodal. Adopting a systems biology approach, embedding‐based algorithms provide a new perspective to examine diseases under a unified framework by mapping diseases into a high‐dimensional space as embedding vectors. These vectors and their constituted disease space encode pathological information and enable a quantitative and systemic measurement of the similarity between any pair of diseases, opening up an avenue for numerous types of downstream analyses. Here, we exemplify its potential through applications in discovering hidden disease associations, assisting in genetic parameter estimation, facilitating data‐driven disease classifications, and transforming genetic association studies of diseases in consideration of comorbidities. While underscoring the power and versatility of this approach, we also discuss the challenges posed by medical context, requirements of online training and result validation, and research opportunities in constructing foundation models from multimodal disease data. With continued innovation and exploration, disease embedding has the potential to transform the fields of disease association analysis and even pathology studies by providing a holistic representation of patient health status.

## 1 INTRODUCTION

Health‐related data are often characterized by several unique features that pose challenges and offer opportunities for analysis. Key features include sparsity, large‐scale, multi‐modality, and others [1]. First, health data are often sparse, containing numerous missing values or zero entries, and this could be due to many practical limitations such as incomplete records, selective data collection, and episodic data entry. Second, health data are typically large‐scale, encompassing vast amounts of information for a large population from diverse sources. Common examples include electronic health records (EHRs) containing detailed medical histories for millions of patients [2, 3, 4], genome‐scale sequencing datasets from various initiatives for hundreds of thousands of individuals [5, 6, 7, 8], and massive repositories of medical images accumulated during every day clinical examinations. Third, health data, exhibiting multi‐modality, encompasses diverse formats, including textual data (e.g., clinical notes, patient histories, and discharge summaries), tabular data (e.g., lab results, medication lists, and vital signs), imaging data (e.g., X‐rays, MRIs, CT scans, and ultrasounds images), genomic data (e.g., sequences and variants from DNA, RNA, and protein analyses), sensor data (e.g., continuous data from wearable devices and medical sensors such as heart rate monitors and glucose sensors), and audio data (voice recordings and other audio data used in diagnosing conditions such as sleep apnea or mental health disorders).

Understanding and making the most use of these features involves dimension reduction and representation learning. In this regard, traditional statistical methods and simple machine learning approaches often struggled to analyze the diverse range of medical data simultaneously, limiting the accuracy of the analysis results. Further, they typically treated diseases as discrete entities without exploring the relationships between them, such as comorbidity, shared risk factors, pathophysiological pathways, and phenotypic overlaps. Adopting a systems biology approach, recent technical advancements aimed to address the challenges of disease data analysis by introducing disease embeddings. Rather than working with the original data, researchers mapped clinical records to a continuous, high‐dimensional, metric‐based disease embedding space, a process which preserves the original semantic meaning of original data. They assumed that the statistical properties of disease are similar to those of language [9] and hypothesized that diseases described in close clinical contexts tend to have closer relationships, which can be represented as linear transformations in the embedding space.

Word embedding algorithms were adopted to implement the concept of disease embeddings. It is based on the distributed representation hypothesis [10] that posits that words appearing in similar contexts tend to have similar meanings. These algorithms leverage neural networks to encode words as numerical vectors in a continuous space, wherein semantically similar words are mapped to nearby regions, and the degree of similarity between words is reflected by the metric distance between their corresponding vector representations.

Several word embedding algorithms, such as Word2Vec [11], GloVe (Global Vectors for Word Representation) [12], FastText [13], BERT (Bidirectional Encoder Representations from Transformers) [14], and ELMo (Embeddings from Language Models) [15], have proven effective in representing words. Word2Vec is a popular word embedding technique developed by researchers at Google. It includes two model architectures: Continuous Bag of Words and Skip‐gram. GloVe, developed by researchers at Stanford, focuses on word‐level similarities in a static context, using either local window‐based or global matrix factorization approaches to learn embeddings. FastText, developed by Facebook’s AI Research lab, extends Word2Vec by representing each word as a bag of character *n*‐grams. This allows it to handle out‐of‐vocabulary words better than other models. BERT, developed by Google, provides a dynamic, context‐aware representation for each word based on the other words in the sentence, utilizing the powerful Transformer [16] architecture and a combination of unsupervised learning tasks. ELMo, developed by researchers at the Allen Institute for AI, generates context‐aware word embeddings using a bidirectional LSTM (Long Short‐Term Memory) [17] model trained on a language modeling objective.

Recent medical embedding models such as Med‐BERT [18], BEHRT [19], REGLE [20], and Med‐Gemini [21] apply deep learning to enhance healthcare data analysis. Med‐BERT and BEHRT adapt the BERT and Transformer architecture for clinical tasks. REGLE, a representation learning model for gene discovery in low‐dimensional embeddings, is introduced to uncover associations between genetic variants and high‐dimensional clinical data. While Med‐Gemini is a robust multimodal model suite for medical applications, seamlessly integrating web search and offering easy customization for new data types through specialized encoders. Together, these models advance precision medicine and personalized healthcare by capturing complex relationships in medical data. We summarized the methods mentioned, along with their medical applications, pros, and cons in the Table 1.

**TABLE 1:** Overview of medical embedding models, description, pros, and cons.

Model name	Description	Pros	Cons	Reference
Word2Vec	Disease classification, electronic health record (EHR) data analysis, and feature extraction for other tasks	Simple and efficient, captures word‐level similarities	Context‐independent, limited to static embeddings	[11]
Med‐BERT	Disease prediction with EHR data, effective with limited training data	High accuracy, low data requirements, accelerates AI in healthcare	High training cost, complex, and overfitting risk	[18]
BEHRT	Predicting 301 diseases, early detection, personalized medicine, and feature extraction for other tasks	High accuracy, scalable, personalized interpretation, supports transfer learning	High data and computation needs, limited interpretability, and generalization concerns	[19]
REGLE	Discovering genetic variants from high‐dimensional clinical data (HDCD) for improving genomic discovery and disease prediction	Unsupervised learning, captures complex features, improves GWAS/PRS accuracy, identifies clinically relevant features, and more efficient than traditional GWAS	High computational resource needs, limited generalization across datasets, “Black box” interpretability, relies heavily on data quality, potential overfitting	[20]
Med‐Gemini	Clinical reasoning, multimodal data understanding, long‐text processing (EHR, medical videos)	High clinical reasoning accuracy (91.1%), strong multimodal performance, handles long texts, real‐time utility, integrated web search, and adaptable to new data types	Data bias, potential errors in multimodal data, safety concerns in clinical use, slower with long texts, bias in external information, limited transparency, and regulatory challenges	[21]

*Note*: This table provides a concise summary of the key medical embedding models discussed in the manuscript. It highlights their specific applications in healthcare, as well as the associated advantages and limitations of each model.

Jia et al. [22, 23] mapped clinical records of over 151 million unique Americans to a computable and continuous high‐dimensional disease embedding space using word‐embedding inspired algorithms [24]. To compute disease embeddings, they substituted natural language words with disease groups, represented sentences as chronological sequences of patient‐specific disease groups, and replaced the text corpus with a large collection of patient‐specific diagnosis records. This allows for the analysis of diseases at a systemic level, revealing hidden relationships between diseases. Their study goes beyond merely clustering diseases using embedding techniques; they introduced disease embeddings as novel phenotypic traits and, through genome‐wide association studies (GWAS), identified genetic associations linked to these newly defined disease traits. By leveraging data from the UK Biobank, BioBank Japan, and BioVU for cross‐validation of their GWAS findings, Jia et al. not only applied cutting‐edge embedding techniques but also broke new ground by linking disease relationships with underlying genetic data.

They employed the Gensim [25] package to train the disease corpus and obtained a 20‐dimensional disease embedding space. Each disease is represented by a 20‐dimensional vector, denoted as $\left\{E_{1}^{w},E_{2}^{w},\ldots ,E_{20}^{w}\right\}$. The cost function used in disease embedding is customized as shown in Equation (1).

(1) $$\begin{array}{c} \text{cost}=-\sum_{\omega \in C}\log\;P\left(\omega |\omega_{-}\right). \end{array}$$

Individual diseases were denoted as *ω* and co‐occurring diseases as *ω*_, which can be considered as contextual information related to a disease in a patient’s diagnosis record. The diagnosis record comprises 547 disease groups, and all disease‐related information is stored in the corpus C. The objective of the cost function is to maximize the probability of a disease given its context by minimizing the negative log likelihood, as implemented in Word2Vec.

**FIGURE 1:**
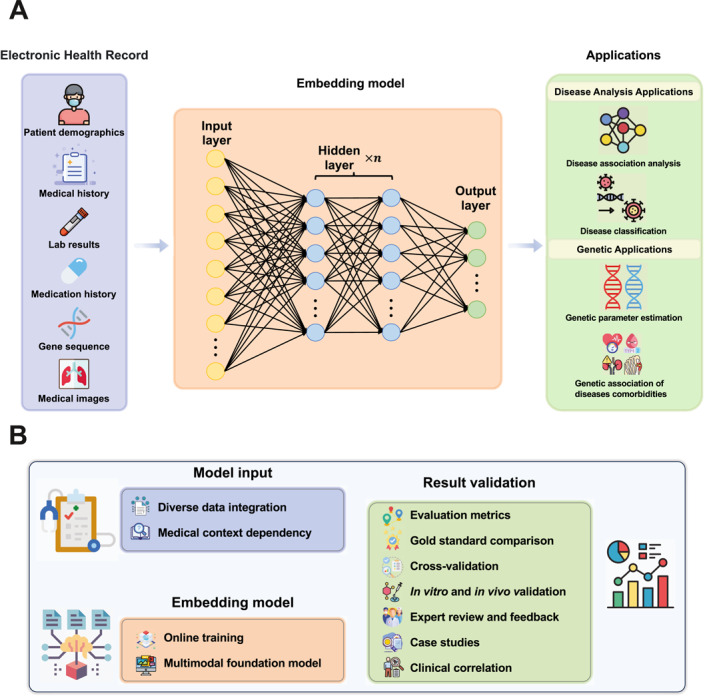
Disease embedding workflow and challenges. (A) Disease embedding workflow. The process of disease embedding involves several steps, starting from the collection of electronic health records (EHRs) to the application of the resulting embeddings in various disease analysis tasks. Here is a general overview of the workflow: (1) *Data collection*: The first step in the disease embedding process is the collection of EHRs. These records contain a wealth of information about patients, including demographics, medical history, lab results, medication history, gene sequences, and medical images. These data serve as the raw material for the disease embedding process. Once the data are collected, they need to be preprocessed to prepare them for the embedding process. This can involve cleaning the data, dealing with missing values, normalizing numerical data, and encoding categorical data. (2) *Disease embedding*: After preprocessing, the data are fed into an embedding model, such as a neural network. This model maps each disease to a high‐dimensional vector that captures the disease’s characteristics and relationships with other diseases. (3) *Applications*: Finally, the disease embeddings can be applied in various disease analysis tasks. These can include: disease association analysis, data‐driven disease classification, genetic parameter estimation, and disease comorbidity analysis, among others. Moreover, the potential applications of disease embeddings extend beyond these areas, with a wealth of opportunities awaiting exploration by researchers in the field. (B) Challenges and outlook of disease embedding. While disease embedding has shown significant potential in transforming the field of disease analysis, it also presents several challenges that need to be addressed: (1) *Model input challenges*. Integrating diverse data sources, such as ICD‐9 and ICD‐10, complicates standardization, while incomplete and imbalanced records degrade embedding quality. Missing data, whether random or systematic, introduce bias, and traditional imputation methods fail to capture complex medical relationships. Cross‐domain integration, such as genomic or environmental data, is further hindered by differing semantics and privacy regulations. Additionally, EHR data pose challenges such as polysemy (terms with multiple meanings) and synonymy (different terms for the same condition), while embedding models struggle with context dependency, where term meaning is influenced by the surrounding context, affecting interpretation. (2) *Embedding model challenges*. As new health data are constantly being generated, disease embedding models need to be able to update their embeddings in an online, or incremental, manner. This requires models that can learn from new data without having to be retrained from scratch. Many existing models demonstrate proficiency in handling text data, yet they often encounter challenges when tasked with processing multimodal data, such as medical images or genetic sequences. This highlights the urgent necessity for the development and implementation of multimedia foundation models. These models, designed to effectively manage diverse data types, would significantly enhance the accuracy and efficiency of disease embeddings, advancing the field of medical data analysis. (3) *Result validation challenges*. To ensure the authenticity of the results derived from disease embeddings, it is essential to employ a variety of validation methods. This begins with the utilization of quantitative measures, which assess the performance and accuracy of the embeddings. The results are then compared with established “gold standard” datasets or results within the field, serving as a benchmark for quality and accuracy. Cross‐validation improves the results by increasing reliability and robustness, as it helps validate the findings and reduce overfitting. Conducting in vitro (laboratory) and in vivo (live organism) experiments provide a real‐world context for the validation of the disease embeddings. Expert reviews and feedback are also invaluable, offering critical insights and suggestions for improvement. The application of disease embeddings to specific case studies allows for an examination of their effectiveness and accuracy in real‐world scenarios. Finally, correlating the results of disease embeddings with clinical outcomes serves to validate their predictive power, further confirming their utility in the field of medical data analysis.

The disease embedding space provides a continuous and high‐dimensional representation for each disease, enabling researchers to analyze and compare different diseases within a unified framework. In this space, diseases are mapped as points, and their positions reflect the pathological similarities and differences among diseases. It is demonstrated that this embedding space and its coordinates can encode pathology‐related information, as evidenced by its wide applications. Here, we elaborate on four use cases of disease embedding as examples. Figure 1A provides a visual summary of the disease embedding model’s workflow and its applications.

## 2 DISEASE EMBEDDING IN DISEASE ANALYSIS APPLICATIONS

In this section, we explore how disease embeddings can enhance our understanding of disease relationships and improve analytical methodologies.

### 2.1 Comparing diseases and uncovering hidden disease associations

Elucidating complex relationships among diseases and identifying novel disease associations are crucial aspects of disease analysis, with implications for diagnosis, treatment, and understanding disease etiology. Recent research has focused on leveraging advanced techniques, including network analysis [26], machine learning [27], and more recently, disease embeddings [23].

First, network‐based approaches represent diseases as nodes and disease associations as edges in a graph structure. By analyzing the topology of disease networks, researchers can identify communities of related diseases. However, the binary nature of these networks—representing either the presence or absence of associations between diseases—limits their ability to capture the nuanced similarities necessary for a deeper understanding of disease relationships.

Second, machine learning models trained on large‐scale healthcare data and leveraged features such as patient demographics, medical history, and genetic information to identify patterns and relationships among diseases. However, these models require labeled data and their interpretability remains limited, making it difficult to translate findings into biological mechanisms or disease pathways. Additionally, their reliance on predefined features constrains the discovery of novel associations.

Third, disease embeddings enable researchers to quantify disease similarity by encoding the diverse attributes of diseases into a unified vector representation and measuring the geometric distance between disease vectors in the embedding space. Unlike traditional models, embeddings automatically learn feature representations from data without the need for labeled training sets. They integrate diverse data sources—such as clinical, molecular, and epidemiological information—enabling the capture of more complex and subtle disease relationships. Diseases that are closer in the embedding space tend to share more similar characteristics, including pathophysiological features, clinical presentations, molecular profiles, therapeutic responses, and epidemiological associations. Conversely, those that are further apart suggest greater dissimilarity, potentially reflecting distinct underlying mechanisms, clinical presentations, or molecular characteristics. Researchers quantified disease proximity by calculating the cosine similarity between disease‐specific vectors, effectively representing the relationships as angles in a high‐dimensional space. Consequently, modeling diseases as high‐dimensional points enables a quantitative and systemic measurement of the similarity between any pair of diseases and allows to identify the antipode (most distant disease) for each one.

The key advantage of disease embeddings lies in their ability to uncover latent connections, unnoticed patterns, and associations between diseases that may not be immediately apparent from traditional diagnostic or classification schemes. Exploiting the capacity of Word2Vec to capture linguistic regularities and patterns within word embedding vectors [11], it is plausible to anticipate that employing a similar methodology can generate disease vectors capable of capturing higher‐order etiological relationships between diseases. This capability facilitates the computational discovery of hidden disease analogies, with relationships between diseases represented as linear transformations. For instance, by representing each disease as a 20‐dimensional vector, researchers can utilize vector algebra to reveal approximate relationships between disease vectors [23]. It is worth noting that researchers observed that (chondrocalcinosis) + (connective tissue infection) is approximately equivalent to (septic arthritis), and (abnormal spine curvature) − (congenital spine anomaly) + (gout related crystal arthropathies) is approximately equivalent to (chondrocalcinosis). These established disease analogies can carry potentially informative implications for comprehending the combinatorial properties inherent in complex diseases.

Human Phenotype Ontology (HPO) plays a crucial role in rare disease research by providing a standardized vocabulary for phenotypic abnormalities, which is essential for accurate diagnosis and genotype–phenotype correlations. Integrating disease embeddings with HPO can further enhance the understanding of rare diseases by identifying hidden associations between phenotypes and rare genetic conditions. This integration could improve phenotype‐driven diagnostics and facilitate the discovery of novel disease subtypes, thereby advancing the field of rare disease research.

Using the embedding algorithm to analyze HPO data improved the accuracy of phenotype‐driven diagnostics by providing a more efficient approach to capturing the complex biological and clinical relationships encoded in HPO terms. Compared to traditional methods such as Resnik, the embedding‐based approach offers faster computation and greater efficiency, particularly for downstream tasks such as patient similarity analysis [28].

### 2.2 Facilitating data‐driven disease classification

Disease classification is a fundamental task in healthcare that measures disease associations systemically. Traditional methods of disease classification typically rely on clinical signs and symptoms, medical history, laboratory tests, medical imaging, and very importantly, the expertise and experiences of healthcare professionals [29]. Although these approaches remain valuable in clinical practice, they may be limited by subjectivity, variability in interpretation, and the complexity of certain diseases. Emerging technologies such as artificial intelligence offer opportunities to enhance disease classification by automating diagnostic processes, integrating multimodal data, and improving diagnostic accuracy. Disease embedding space offers a data‐driven approach to disease classification. By distilling multifaceted disease attributes such as clinical symptoms, genetic predispositions, and epidemiological patterns into vector representations, diseases can be analyzed and clustered directly using machine learning and statistical methods. Algorithms such as *k*‐means clustering and hierarchical clustering [30, 31] can identify natural groupings of diseases based on their vector positions, revealing novel insights into disease classification unconstrained by preexisting taxonomic structures.

Disease embeddings enable researchers to gain deeper insights into the classification relationships among different diseases, potentially uncovering novel disease subtypes or comorbidity pairs [23]. Researchers performed the singular value decomposition algorithm on disease embedding space and obtained 10 maximally distinct disease constellations. They assigned diseases to different constellations by calculating the cosine similarity between each disease and each constellation. For instance, in the International Classification of Diseases (version 9; ICD‐9), a globally recognized coding system used to classify diseases and health conditions for statistical purposes, migraine is classified alongside eye inflammation within the “diseases of the CNS and sensory organs” category (ICD‐9 codes 320–389). However, data‐driven disease classification studies suggested that migraine may be more closely related to immune system diseases [23, 32].

Jiang et al. employed disease embedding to encapsulate medical knowledge, establishing relationships between symptoms and disease entities [33]. They integrated disease embedding into a recursive neural knowledge network (RNKN). The 200‐dimensional disease embedding vector, derived from the RNKN, exhibits clustering properties that are largely consistent with the ICD code. This suggests that diseases within the same category tend to cluster together in the embedding space, providing further validation of the effectiveness of the data‐driven classification approach within the disease embedding space.

## 3 DISEASE EMBEDDING IN GENETIC APPLICATIONS

Expanding beyond disease analysis, we also examine the role of disease embeddings in genetic studies, where they contribute to a deeper understanding of genetic influences on health.

### 3.1 Assisting in genetic parameter estimation

Genetic parameters, such as heritability, and genetic correlations are crucial for us to understand the pathology and genetic foundation behind disease associations. Traditional estimation approaches include comparing disease patterns among monozygotic and dizygotic twins [34], analyzing nuclear family phenotypic variance [35] and utilizing GWAS outputs [36]. These estimation methods require substantial genetic, pedigree, and phenotypic information to work.

Fortunately, the efficacy of the accumulating legacy estimates of genetic parameters contributes to the provision of a novel approach for estimation. The utilization of disease embeddings transcends the limitations of traditional genetic analysis by providing a holistic representation of disease traits [23]. Rather than focusing solely on individual genetic variants or phenotypic traits, disease embeddings encapsulate a comprehensive spectrum of disease characteristics, ranging from clinical manifestations to molecular signatures. This view enables researchers to consider the intricate interrelationships between diverse disease features and their genetic underpinnings, enriching the genetic parameter estimation process.

Researchers integrated disease embeddings into genetic parameter estimation, representing a paradigm shift in the study of complex diseases. They utilized disease embeddings and disease prevalence curves that reflect the multiplicity of age‐specific landmark events in a patient’s life as machine learning features and trained mathematical models to predict heritability and genetic correlations in the absence of genetic data [22]. The heritability estimation using gradient boosting regression achieved a Pearson’s correlation value of 0.870 ± 0.001 (95% confidence interval) for heritability prediction. Testing on an independent dataset of genetic correlations [37] demonstrated results that are highly consistent with traditional methods, including the additive and environmental (AE) model, additive, common, and environmental model, polygenic risk score calculations, sequential oligogenic linkage analysis routines, genomic‐relatedness‐based restricted maximum likelihood, and linkage disequilibrium score regression (LDSC). The comparison with LDSC yielded a Pearson correlation coefficient of 0.73 and a Student’s *t*‐test *p*‐value of 1.7 × 10^−14^. These studies have thus showed that the disease embedding vectors were indeed informative to genetic association. Using vectors generated by embedding algorithms as machine learning features opens up new opportunities for predicting various biologically relevant parameters (gene association [38], drug repositioning [39], protein–protein interaction [40]), and predicting genetic parameters demonstrated here are just examples.

### 3.2 Transforming genetic association studies of diseases comorbidities

GWAS of diseases typically analyze single diseases. As many diseases tend to co‐occur and one disease may alter the clinical symptoms, prognoses, and characteristics of another, the lack of considering comorbidity complexity can hinder researchers from elucidating disease etiology and designing effective treatment. As a remedy, disease embedding encapsulates complex similarities among diseases and encompasses all diseases, providing a novel perspective on pleiotropy. Researchers can employ disease embedding vectors as novel phenotypes to apply conventional genetic analysis methods, such as genome‐wide association analysis [23, 41], to investigate the genetic architecture of diseases.

When disease embedding vectors are treated as novel phenotypes, they encapsulate an individual’s overall health status and reflect the similarities and co‐occurrence patterns of multiple diseases. Consequently, genetic associations identified through GWAS are not restricted to a single disease but may extend to several related conditions. Interpreting these findings involves emphasizing how these genetic associations traverse distinct disease clusters, providing valuable insights into comorbidities and the shared genetic basis of diseases.

Researchers used the UK Biobank cohort, comprising approximately half a million participants, to conduct a genome‐wide association study on disease embedding vectors that represent individuals’ overall health conditions [23]. The analysis of disease embedding and genetic associations contributes to predicting individual susceptibility and risk for specific diseases. They discovered 116 genetic associations involving 108 genetic loci and then used 10 disease constellations resulting from clustering analysis of diseases in the embedding space, as well as 30 common diseases, to demonstrate that these genetic associations can robustly predict various morbidities.

## 4 CHALLENGES AND OUTLOOK

Disease embeddings, which represent diseases in a continuous vector space, offer the promise of enhanced research into disease relationships and deeper exploration of pathological mechanisms. However, realizing this potential is not without its obstacles. There are several challenges associated with model input, embedding model capability, and result validation, as illustrated in Figure 1B.

### 4.1 Limitations of the medical data

One of the key limitations of medical data is the challenge of integrating diverse data sources. Differences in data formats and coding standards, such as the use of ICD‐9 versus ICD‐10 or varying representations of medications, make standardization difficult, potentially affecting the quality of disease embeddings. Additionally, the level of detail recorded varies across datasets, with some providing comprehensive histories and others offering limited information, leading to asymmetry and incomplete embeddings. Integrating cross‐domain knowledge, such as genomic or environmental data, further complicates this process due to differing semantics, whereas strict privacy regulations restrict data sharing across institutions, limiting access to critical patient records and reducing the comprehensiveness of the embeddings.

Medical data often contains substantial missing information, posing a significant challenge for generating reliable disease embeddings. Missing data can be random or systematic, with the latter introducing bias that affects embedding accuracy—for example, certain hospitals may not record specific tests or patient histories may lack critical details. Common imputation methods, such as mean filling, regression, or interpolation, often fail to capture the complexity of medical relationships and may introduce spurious correlations, distorting the model’s learning of true disease associations. Moreover, data imbalance further complicates embedding quality, as common diseases are well‐represented while rare conditions often suffer from insufficient data, exacerbating model performance disparities.

Disease embeddings are deeply influenced by the medical context in which they are derived. In the medical world, “context” refers to the comprehensive environment or setting in which a health condition, medical practice, or health‐related event occurs. This context can significantly influence the understanding, diagnosis, treatment, and progression of diseases. However, the assumptions underpinning traditional word embeddings can falter due to the complexity and specificity inherent in medical data. One notable challenge is medical ambiguity and polysemy [42], where terms may have multiple meanings depending on the context. For instance, “cold” might denote a common viral infection or describe low body temperature in hypothermia scenarios. This ambiguity can lead to misinterpretation and errors in automated text analysis, particularly in distinguishing between similar terms or resolving semantic ambiguities.

Synonymy presents an additional challenge, where different expressions of disease names may refer to the same disease. Mapping all synonyms into the same unified name requires substantial knowledge of the specific medical domain and elaborative manual calibration. Other issues are related to unstructured nature, data quality and completeness, and variability in language conventions.

Another limitation lies in the context dependency, which overlooks crucial long‐range dependencies in medicine. For example, childhood mental health issues can have implications for health in adulthood [43], extending beyond the local context window used in word embedding models. These long‐range dependencies are critical for understanding the full impact of medical conditions and treatments, yet traditional embedding approaches inadequately captured them.

External medical knowledge resources, such as medical dictionaries or ontologies, can provide valuable term definitions and related information to address medical polysemy and synonymy. Resources such as UMLS (Unified Medical Language System) [44], MeSH (Medical Subject Headings) [45], SNOMED CT (Systematized Nomenclature of Medicine—Clinical Terms) [46], and RxNorm [47] offer essential information and structure to disambiguate medical terms, enhancing the accuracy and consistency of disease embeddings and effectively managing medical synonymy. In cases where specific polysemous term issues persist, manual annotation by domain experts becomes necessary. This process ensures the correct capture of term semantics during the embedding process, further refining the effectiveness of disease embeddings in reflecting medical context.

Although disease embeddings from medical data have the potential to capture complex relationships between diseases, significant research limitations persist, particularly in data integration and handling missing information. To overcome these challenges and enhance embedding performance, advanced approaches such as multimodal learning and specialized imputation models are necessary. Additionally, developing robust natural language processing techniques, standardized terminologies, and interoperable data frameworks is crucial to improving the accessibility, usability, and interoperability of medical text data for both research and clinical applications. Safeguarding data privacy, ensuring quality control, and addressing ethical concerns are essential to maintaining patient confidentiality and trust in healthcare data analytics.

### 4.2 Online training of disease embedding

Online training of disease embeddings refers to the continuous and dynamic learning of vector representations of diseases, allowing them to adapt to newly gained data and growing knowledge. Medical knowledge is evolving with the emerging new diseases and the discovery of new connections between existing ones. Healthcare data, such as EHRs and research publications, is often generated in a streaming fashion. Online training can incorporate this data as it arrives, eliminating the need for retraining from scratch and enabling timely updates. Online training can personalize disease embeddings for individual patients, considering their specific medical history and context, leading to more accurate and personalized healthcare insights.

Here are some key points of online training methods for disease embedding. (1) *Streaming Data Processing* [48]: Process healthcare data streams in real‐time or in batches, extracting disease‐related information from EHRs, medical literature, genetic databases, and other sources. Apply online learning algorithms to update disease embeddings on‐the‐fly as new data points are received. (2) *Online learning algorithms*: Online learning algorithms are crucial for managing and analyzing large‐scale healthcare datasets, ensuring that models remain robust and accurate with streaming data sources. Online gradient descent [49] is a fundamental technique where model parameters are iteratively updated based on the gradient of the loss function, enabling real‐time learning with minimal computational overhead, which is essential for processing vast amounts of healthcare data efficiently. Adaptive regularization of weight vectors [50] enhances robustness by dynamically adjusting regularization, thereby maintaining a balance between stability and adaptability in the presence of noisy and heterogeneous healthcare data. Follow‐the‐leader (FTL) [51] selects the best‐performing model based on historical performance, which can be effective but may encounter instability in non‐stationary healthcare environments. To address this, follow‐the‐regularized‐leader (FTRL) [43] incorporates regularization into the FTL framework, providing a more stable and resilient approach by penalizing large updates and smoothing the learning trajectory. Collectively, these algorithms are indispensable for the continuous refinement and adaptation of models in large‐scale healthcare datasets, enabling timely and accurate insights critical for patient care and medical research. (3) *Incremental Learning* [52]: Instead of retraining the entire embedding model from scratch, incremental learning techniques adjust the embedding vectors based on incoming data samples while preserving the previously learned knowledge. (4) *Adaptive Models* [53]: Develop adaptive disease embedding models that dynamically adjust their parameters and embeddings in response to changes in data distribution, patient populations, or clinical practices. Adaptive models incorporate feedback mechanisms to incorporate new knowledge, refine embeddings, and maintain model performance. (5) *Reinforcement Learning* [54]: Explore reinforcement learning techniques to train disease embedding models in online settings, where the model interacts with the environment (e.g., healthcare system) and receives feedback (e.g., patient outcomes) to update embeddings. Reinforcement learning frameworks enable adaptive learning and decision‐making in dynamic healthcare domains. (6) *Dynamic Embedding Spaces* [55]: Design disease embedding spaces that can expand or contract based on the complexity and diversity of healthcare data. Dynamic embedding spaces accommodate new disease entities, and emerging medical concepts, ensuring the scalability and flexibility of disease embedding models.

### 4.3 Foundation models built on multimodal disease data

The etiology of diseases is often complex [56], with their onset commonly attributed to a multifaceted interplay of genetic, environmental, lifestyle, biological, and socioeconomic factors. To understand and address disease information comprehensively, algorithms employed for generating disease embedding vectors should be capable of analyzing vast amounts of heterogeneous data from diverse sources, such as EHRs, biomedical literature, genetic databases, clinical images, and audios.

A foundation model might be a suitable choice for generating disease embeddings by analyzing these diverse data. Foundation models [57] present a new paradigm in artificial intelligence, referring to very large AI models trained on massive amounts of data. These models are designed to be versatile and adaptable, serving as a foundation for various downstream tasks [58]. They learn general representations and patterns from the data, enabling them to be fine‐tuned for specific applications.

Several foundation models, such as BioBERT [59], Med‐PaLM 2 [60], BioELECTRA [61], and BioBART [62], have been developed specifically to decipher the biological information embedded within textual data related to DNA, RNA, and proteins. There is an urgent need to develop a multimodal disease foundation model that can integrate diverse data modalities such as textual information, images, audio, genetic data, and clinical records. Heterogeneous data sources offer diverse viewpoints on a patient, providing valuable insights that enhance support for a wide range of clinical decisions [63]. Developing a multimodal disease foundation model can facilitate personalized medicine and improve clinical decision‐making by integrating and analyzing comprehensive patient data, leading to more accurate diagnoses, treatments, and outcomes.

### 4.4 Disease embedding result validation

To ensure that the results from disease embedding spaces reflect real biological or clinical phenomena rather than being artifacts of mathematical procedures, researchers must validate their findings and here are some approaches.

(1) *Evaluation metrics*: Define appropriate evaluation metrics to assess the quality of disease embeddings. Common metrics include cosine similarity [64], Euclidean distance [65], and clustering performance measures such as silhouette score [66] or adjusted Rand index [67]. These metrics quantify how well the embeddings preserve semantic relationships and group similar diseases together. (2) *Gold standard comparison*: Compare disease embeddings with established gold standards or reference datasets, such as disease ontology hierarchies (e.g., MeSH) or expert‐curated disease similarity rankings. Evaluate whether the embeddings reflect known disease relationships and exhibit concordance with established disease classifications or taxonomies. (3) *Cross‐validation*: Perform cross‐validation to assess the robustness and generalizability of disease embeddings across different datasets or data partitions. Divide the data into training and validation sets, train the embedding model on the training set, and evaluate its performance on the validation set to ensure that the embeddings generalize well to unseen data. (4) In vitro and in vivo *experiment*: researchers should examine specific biological contexts to understand the impact of genes or pathways highlighted by disease embeddings. This process often entails manipulating cellular models, such as cell cultures, and employing techniques such as gene expression analysis or biochemical assays. Through these experiments, researchers can observe and measure the effects of the identified genes or pathways on disease‐related processes, thus confirming the validity of biology informed by the disease embedding spaces. In vivo analyses are also essential to confirm the applicability of disease embedding findings at the level of the whole organism. Researchers can use animal models, such as genetically modified mice or other suitable organisms, to examine the phenotypic consequences of genetic or molecular modifications suggested by disease embeddings. (5) *Expert review and feedback*: Seek feedback from domain experts, clinicians, and biomedical researchers to validate the interpretability and clinical relevance of disease embeddings. Engage experts in reviewing the embeddings, assessing their clinical validity, and providing qualitative feedback on their utility for medical decision‐making and research applications. (6) *Case studies*: Conduct case studies or use cases to show the practical utility of disease embeddings in real‐world healthcare scenarios. Show how disease embeddings can facilitate tasks such as disease prediction, patient stratification, treatment recommendation, or cohort identification, and validate their effectiveness through empirical validation studies. (7) *Clinical correlation*: Validate disease embeddings by assessing their correlation with clinical outcomes, disease severity, treatment responses, or other relevant clinical parameters. Investigate whether diseases that are closer in embedding space exhibit similar clinical profiles or share common risk factors, genetic pathways, or comorbidities.

By employing a combination of these validation approaches, researchers can ensure the robustness, validity, and clinical relevance of disease embeddings, ultimately enhancing their utility for advancing biomedical research and improving healthcare outcomes.

## 5 CONCLUSION

In representation learning applied to disease association studies, disease embedding models serve as powerful frameworks for capturing complex relationships within heterogeneous, multimodal health data. These data, often sparse and large‐scale, present challenges in analysis, and embedding algorithms offer a promising solution for dimension reduction and information extraction. By representing diseases as quantitative vectors, the models facilitate the measurement and understanding of intricate disease relationships, thus supporting more informed and efficient medical decision‐making.

The future of disease embeddings holds exciting potential for transforming healthcare research. One promising direction is the integration of disease embeddings with real‐time clinical decision support systems, enabling personalized patient care through the identification of comorbidities and disease progression patterns. Additionally, as multi‐omics data (such as proteomics, metabolomics, and epigenomics) become more accessible, embedding models could expand to incorporate these data types, offering a more comprehensive view of disease mechanisms and genetic interactions. Embedding models could also be applied to precision medicine initiatives, helping to tailor treatments based on individual disease profiles and genetic data.

Moreover, the increasing availability of longitudinal EHR data presents opportunities for embedding models to capture temporal disease dynamics more accurately. Future advancements may involve integrating these models with deep learning architectures that specialize in time series data, such as recurrent neural networks or transformers, to improve predictions of disease trajectories and outcomes.

## AUTHOR CONTRIBUTIONS

**Tianxin Xu**: Investigation; visualization; writing—original draft; writing—review and editing. **Yu Li**: Writing—review and editing. **Xin Gao**: Writing—review and editing. **Andrey Rzhetsky**: Writing—review and editing. **Gengjie Jia**: Conceptualization; funding acquisition; investigation; project administration; supervision; writing—review and editing.

## CONFLICT OF INTEREST STATEMENT

The authors declare no conflicts of interest.

## ETHICS STATEMENT

There was no sample from human subjects or animals collected for this work.

## Data Availability

Data sharing is not applicable to this article as no new data were created or analyzed in this study.
